# Gel immersion transpapillary stone removal for recurrent choledocholithiasis

**DOI:** 10.1055/a-2715-5094

**Published:** 2025-10-29

**Authors:** Yota Hirayama, Tesshin Ban, Kei Ando, Naoto Imura, Yoshimasa Kubota, Shun Sasoh, Takashi Joh

**Affiliations:** 1 36884 Department of Gastroenterology and Hepatology, Gamagori City Hospital, Gamagori, Aichi, Japan

Endoscopic retrograde cholangiopancreatography (ERCP)-guided stone removal using a balloon catheter is a promising approach for patients with small choledocholithiasis (diameter: ≤10 mm 1 2 ). The recurrence of choledocholithiasis significantly increases in patients with certain characteristics, such as dilated common bile duct (CBD), advanced age, history of cholecystectomy, and multiple stones 3 . However, data on endoscopic strategies for the frequent recurrence of choledocholithiasis, debris, or sludge formation are scarce. Gel immersion endoscopy has emerged as a novel technique and has also been reported in ERCP-related procedures 4 5 . Here, we report the potential of gel immersion ERCP-guided clearance for recurrent choledocholithiasis.

A 94-year-old woman with a history of ERCP-guided stone removals presented with acute cholangitis due to a suspected third recurrence of choledocholithiasis. ERCP-guided stone removal was attempted using a balloon catheter (Extractor Pro; Boston Scientific, Marlborough, USA); however, only the mucous content was removed ( Fig. 1 **a, b** , Video 1 ). Next, we inserted an endoscopic introducer (EndoSheather; Piolax Medical Device, Yokohama, Japan) into the CBD, followed by bile aspiration and injection of the 10 mL gel (Viscoclear; Otsuka Pharmaceutical Factory, Naruto, Japan) with contrast medium. This maneuver facilitated the removal of small residual stones and debris without adverse events ( Fig. 2 **a, b** , Video 1 ).

**Fig. 1:**
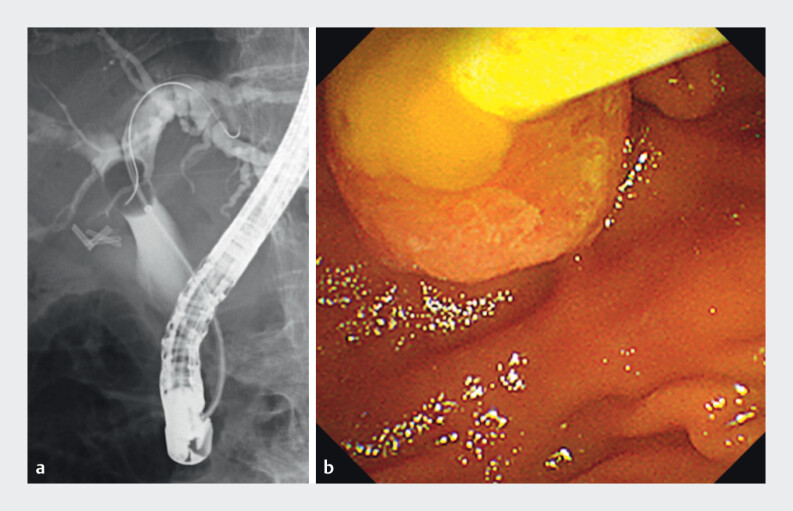
Conventional endoscopic retrograde cholangiopancreatography (ERCP)-guided stone removal using a balloon catheter. **a** Cholangiography is performed during biliary sweep using an inflated balloon. **b** In an endoscopic view, the sweep with a balloon catheter following the injection of a contrast medium results in the extraction of only mucinous contents.

**Fig. 2:**
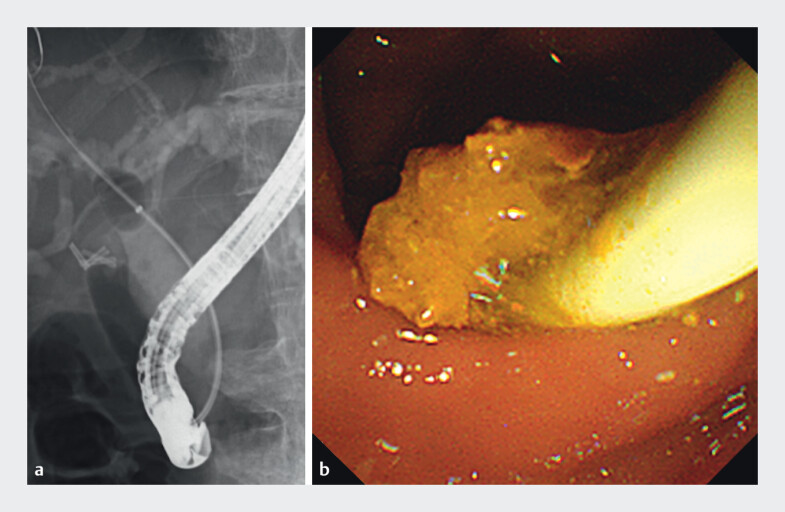
Gel immersion endoscopic retrograde cholangiopancreatography (ERCP)-guided stone removal using a balloon catheter. **a** Cholangiography using a gel with contrast medium. **b** In an endoscopic view, a significant amount of the gel–stone/debris complex is removed.

Gel immersion transpapillary stone removal for recurrent choledocholithiasis.Video 1

The small choledocholithiasis and debris likely escaped through the gap beside the inflated balloon or adhered to the CBD wall during clearance using the balloon catheter ( Fig. 3 , Video 1 ). This indicates the incomplete removal of small stones and debris during conventional ERCP-guided stone extraction. In gel immersion ERCP-guided clearance, small residuals are encased in a highly viscous gel, which may facilitate the extraction of the gel–stone/debris complex while preventing its escape alongside retrieval balloon.

**Fig. 3:**
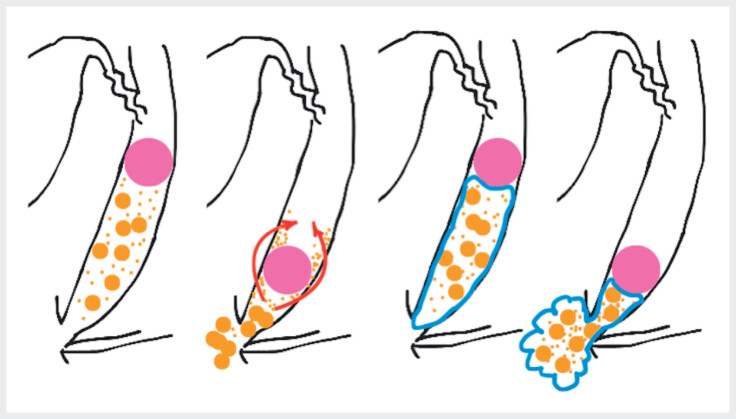
Schema of gel immersion endoscopic retrograde cholangiopancreatography (ERCP)-guided stone removal using a balloon catheter versus a conventional method. In the conventional method, the debris escapes around the balloon (red arrows). In contrast, the debris is removed and enclosed in a highly viscous gel. Left, conventional; right, gel immersion; pink circles, balloons; brown circles, small stones; brown dots, debris; blue areas, gels.

However, further investigation is necessary to address the outcomes, including pancreatitis and recurrence.

Endoscopy_UCTN_Code_TTT_1AR_2AH

## Notes

Endoscopy E-Videos https://eref.thieme.de/e-videos
*E-Videos* is an open access online section of the journal *Endoscopy* , reporting on interesting cases and new techniques in gastroenterological endoscopy. All papers include a high-quality video and are published with a Creative Commons CC-BY license. Endoscopy E-Videos qualify for HINARI discounts and waivers and eligibility is automatically checked during the submission process. We grant 100% waivers to articles whose corresponding authors are based in Group A countries and 50% waivers to those who are based in Group B countries as classified by Research4Life (see: https://www.research4life.org/access/eligibility/ ). This section has its own submission website at https://mc.manuscriptcentral.com/e-videos .
